# Blood pressure variability and global cognition in Black and White older adults over 18 years: a population‐based cohort study

**DOI:** 10.1002/alz.088053

**Published:** 2025-01-09

**Authors:** Anisa Dhana, Charles Decarli, Klodian Dhana, Pankaja Desai, Denis A Evans, Kumar B Rajan

**Affiliations:** ^1^ Rush Institute for Healthy Aging, Chicago, IL USA; ^2^ Rush University Medical Center, Chicago, IL USA; ^3^ University of California, Davis School of Medicine, Sacramento, CA USA; ^4^ Center for Neuroscience, University of California at Davis, Sacramento, CA USA

## Abstract

**Background:**

Variability in blood pressure has been associated with cerebrovascular disease independently of the adverse effects of hypertension or average blood pressure. We investigated whether visit‐to‐visit blood pressure variability is associated with global cognition in older adults.

**Method:**

This study included 4,783 individuals aged 65 and older participating in the Chicago Health and Aging Project, a biracial population‐based study. Blood pressure was measured by a trained research assistant at each study visit, 3 years apart. Blood pressure variability was calculated as the sum of the absolute differences of blood pressure between successive measurements, divided by the number (n‐1) of assessments. Global cognition was assessed by a battery of 4 standardized cognitive tests. Linear regression model was used to evaluate the association of blood pressure variability during the study period with global cognition at the last study visit. The regression model was adjusted for age, sex, race, education, physical activity, cognitive activity, smoking, depression, co‐morbidities (hypertension, heart disease, stroke, and diabetes), baseline cognition, and the time being in the study.

**Result:**

Of 4,783 individuals, 3009 (62.9%) were women, 3146 (65.8%) were Black or African American adults, and the average age of the study population was 71.3 years. Higher systolic and diastolic blood pressure variability was associated with lower cognitive scores. A 1‐SD increase in systolic blood pressure variability was associated with a 0.030 SD lower global cognition (𝛽‐0.030, 95%CI ‐0.055 to ‐0.006). For African American individuals, the 𝛽 estimate was ‐0.040 (95%CI ‐0.069 to ‐0.011), and for White adults, it was ‐0.008 (‐0.052 to 0.036). Similar associations were observed for diastolic blood pressure. In the analysis stratified by antihypertensive medication use, the variability in blood pressure was associated with a lower cognitive score among people who were not on medication, respectively 𝛽 for comparing the 3rd vs. the 1st tertile was ‐0.102 (95%CI ‐0.195 to ‐0.010). There was no association among people who were receiving medication for hypertension.

**Conclusion:**

Elevated blood pressure variability, particularly in older African American adults, is associated with a lower global cognitive score.

